# Delayed intestinal stricture following non-resectional treatment for non-occlusive mesenteric ischemia associated with hepatic portal venous gas: a case report

**DOI:** 10.1186/s12893-015-0028-y

**Published:** 2015-04-08

**Authors:** Shota Maezawa, Motoo Fujita, Takeaki Sato, Shigeki Kushimoto

**Affiliations:** Department of Emergency and Critical Care Medicine/Emergency Center, Tohoku University Hospital, 1-1 Seiryo-machi, Aoba-ku, Sendai, 980-8574 Japan; Division of Emergency and Critical Care Medicine, Tohoku University Graduate School of Medicine, 1-1 Seiryo-machi, Aoba-ku, Sendai, 980-8574 Japan

**Keywords:** Non-occlusive mesenteric ischemia, Hepatic portal venous gas, Delayed stricture, Stenosis

## Abstract

**Background:**

Hepatic portal venous gas associated with non-occlusive mesenteric ischemia is indicative of a serious pathology that leads to bowel necrosis and it has a high mortality rate. Although non-occlusive mesenteric ischemia is acknowledged as a condition that requires early surgical treatment, it has been reported that bowel necrosis and surgical resection of the gangrenous lesion may be avoided if the condition is identified quickly and the cause is detected at an early phase. However, no reports or guidelines have been published that describe the management of patients in whom bowel necrosis and surgical treatment were avoided. We report the case of a patient who presented with non-occlusive mesenteric ischemia who was managed with non-resectional treatment at an early phase and had a delayed small-bowel stricture.

**Case presentation:**

A 24-year-old man presented to the hospital with fever, abdominal pain, and vomiting. Abdominal computed tomography confirmed a diffuse gaseous distention with small-bowel pneumatosis and hepatic portal venous gas. An urgent laparotomy was performed, because septic shock associated with diffuse peritonitis and bowel necrosis was strongly suspected. Although we found purulent ascites and a perforated appendix at the time of surgery, gangrenous and transmural ischemic changes were not evident in the small bowel and colon. We performed an appendectomy without a bowel resection, and the patient was discharged on an oral diet. However, he was re-admitted to hospital, because 4 days after discharge he developed postoperative paralytic ileus. Non-operative management was chosen, but his symptoms did not improve. We decided to perform a laparotomy 40 days after the initial operation, and a considerable adhesion was detected. Therefore, only a synechotomy was performed. On day 57, he experienced symptoms that were associated with bowel obstruction once again. On day 59, a partial resection of the jejunum was performed. Severe luminal strictures were apparent within the jejunum, and marked structural changes were evident.

**Conclusion:**

While non-surgical management can be chosen for selected patients with non-occlusive mesenteric ischemia, continuous observation to evaluate the development of delayed strictures that lead to bowel obstructions is required in patients who undergo non-resectional treatment.

## Background

Hepatic portal venous gas (HPVG) was first described by Wolfe and Evans in infants with necrotizing enterocolitis in 1955 [1]. It has been reported that before the era of computed tomography (CT), the mortality of patients with HPVG that was diagnosed by plain abdominal radiography reached 75% [2]. HPVG is reported to be frequently associated with acute mesenteric ischemia, and this accounts for most of the HPVG-associated mortality. Recently, high-resolution CT has revealed a host of benign conditions that do not require emergency surgery in which the presence of HPVG including abdominal trauma, digestive tract dilation, ulcerative colitis, Crohn’s disease, gastric ulcers, and complications associated with endoscopic procedures. However, the mortality of patients with HPVG-associated bowel ischemia remains high, and it demands immediate surgical intervention [3-6]. HPVG is most frequently associated with bowel necrosis that is caused by acute mesenteric ischemia, for which it has a 43% to 70% incidence, and it is associated with a high mortality rate of up to 75% in the presence of bowel necrosis [7,8].

Non-occlusive mesenteric ischemia (NOMI) is a serious pathology that leads to bowel necrosis, and it is associated with many types of critical condition, including shock, hypovolemia, myocardial infarction, heart failure, aortic insufficiency, renal or hepatic disease, during abdominal or cardiac surgery, and during vasopressor treatment [9-11]. NOMI has a high mortality rate of 45–55%, and an early diagnosis and appropriate surgical treatment are important to improve survival in patients with this condition; however, the early symptoms and characteristics are unclear. In many cases, the disease will have advanced to an irreversible stage before a definitive diagnosis is made [11]. Contrast-enhanced CT scanning reveals patent mesenteric vessels, but the intestinal wall shows a reduced level of enhancement, both of which are indicative of a diagnosis of NOMI. Surgical intervention is required if the ischemic damage progresses in a way that is similar to that in suspected HPVG caused by NOMI [12-15].

On the other hand, it has been reported that bowel necrosis and the surgical resection of gangrenous lesions can be avoided if the condition is identified quickly and the cause is determined at an early stage [8]. Although bowel stenoses that present after non-surgical treatment have been demonstrated in patients with abdominal trauma [16,17], inflammatory enterocolitis, and ischemic enterocolitis [18-20], there are no specific reports or guidelines that describe the management and follow up of patients with NOMI in whom bowel necrosis and surgical treatment have been avoided.

We report the case of a patient with NOMI and HPVG who was managed with non-resectional treatment at an early stage and who developed a delayed small-bowel stricture.

## Case presentation

**Figure 1:**
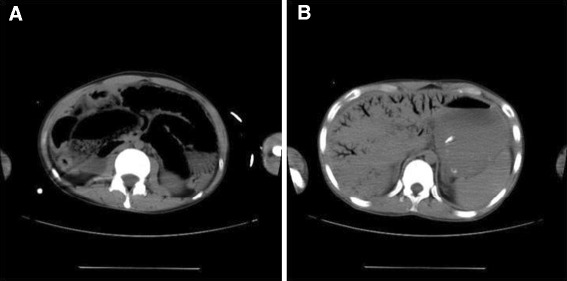
**Computed tomography (CT) findings on the day of admission.** The unenhanced CT scan shows diffuse gaseous distention with pneumatosis of the small bowel **(A)** and extensive hepatic portal venous gas **(B)**.

A 24-year-old Japanese man presented to the emergency department (ED) with fever, abdominal pain, and vomiting. He had a history of mental retardation and epilepsy, and he could not express his emotions verbally. On arrival at the ED, the patient had a blood pressure of 75/30 mmHg, a pulse rate of 160 beats/min with a sinus rhythm, and a respiratory rate of 44 breaths/min. A physical examination revealed that his whole abdomen had a board-like rigidity. Laboratory investigations revealed leukocytosis of 20,600 cells/μl, an elevated C-reactive protein (CRP) level of 28.6 mg/dl, and a procalcitonin level of 278.99 ng/ml (reference range: ≤0.03 ng/ml). Arterial blood-gas analysis showed metabolic acidosis with a pH of 7.411, bicarbonate at 12.6 mmol/l, and lactate at 6.8 mmol/l. A plain abdominal radiograph revealed a diffuse gaseous distention of the small bowel and HPVG. A non-contrast-enhanced abdominal CT scan confirmed the presence of the diffuse gaseous distention that was accompanied by pneumatosis of the small bowel and a dendritic gas image that extended to the liver surface on both the right and left lobes (Figure 1).

**Figure 2:**
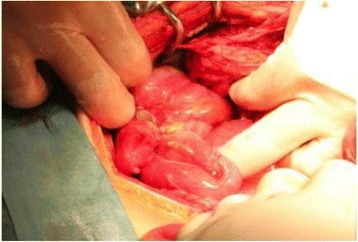
**Intra-operative findings during the initial operation.** Although a perforated appendix was evident, and swelling and serosal inflammatory findings were observed throughout the entire small bowel and colon, gangrenous and transmural ischemic changes were not observed.

Following aggressive fluid resuscitation, an urgent laparotomy was performed because septic shock with diffuse peritonitis caused by perforated appendicitis and bowel necrosis caused by NOMI were strongly suspected. Although we found purulent ascites and a perforated appendix when we explored the abdomen surgically, neither gangrenous nor transmural ischemic changes were observed within the small bowel or colon (Figure 2). Therefore, we performed an appendectomy without a bowel resection and we selected open abdomen management with negative-pressure wound therapy, because a planned re-operation was scheduled. After the initial operation, the patient was admitted to the intensive care unit where he was mechanically ventilated under sedation, and he was required a continuous intravenous infusion of noradrenaline. We administered a meropenem (0.5 g) infusion via a drip 4 times per day. On postoperative day 3, the planned re-operation was performed, and we did not find any ischemic changes within the small bowel or colon. We completed the operation with a formal abdominal closure. His leukocyte, CRP, and procalcitonin levels improved after the initial operation. On postoperative day 9, contrast-enhanced CT was performed, which demonstrated a thickening of the small-bowel wall, but no evidence of bowel ischemia or vascular occlusion. The patient’s blood cultures at the time of admission were positive for *Staphylococcus epidermidis,* and cultures of the intraoperative ascites fluid were positive for *Pseudomonas aeruginosa*, *Escherichia coli*, and *Streptococcus anginosus*, all of which are sensitive to meropenem, which was administered for 14 days. His postoperative course was uneventful, and he was discharged on an oral diet.

Four days after discharge, the patient returned to the ED having experienced several vomiting episodes. A plain abdominal radiograph revealed a partial gaseous distention of the small bowel. Abdominal CT scanning, which was performed on the day following the patient’s readmission to hospital, enabled us to diagnose postoperative paralytic ileus. The patient’s only symptom was vomiting, and he had no symptoms that suggested an abdominal abscess. Non-operative management was chosen that including nasogastric tube decompression. After the discharge from the nasogastric tube declined, we began oral nutrition; however, the patient vomited again and we managed him using a long intestinal tube. The patient’s symptoms did not improve under non-operative management, and we decided to perform a laparotomy 40 days after the initial operation, because an upper gastrointestinal series using a long intestinal tube had revealed a small intestinal stenosis. A considerable adhesion was present in the upper jejunum to the abdominal wall, which were considered to be the cause of the passage disorder, and there was no evidence of a bowel stricture. Based on these intraoperative findings, we performed a synechotomy only.

**Figure 3:**
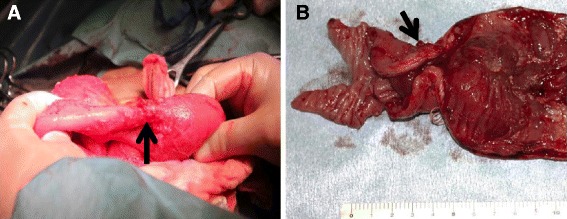
**Intra-operative findings during the third operation.** Marked small-bowel stenosis (black arrow) was evident, which was not observed during the second operation **(A)**. The macroscopic findings of the small-bowel stenosis included a severe luminal stenosis and marked structural changes accompanied by thickening caused by scarring mainly in the luminal side (black arrow) **(B)**.

**Figure 4:**
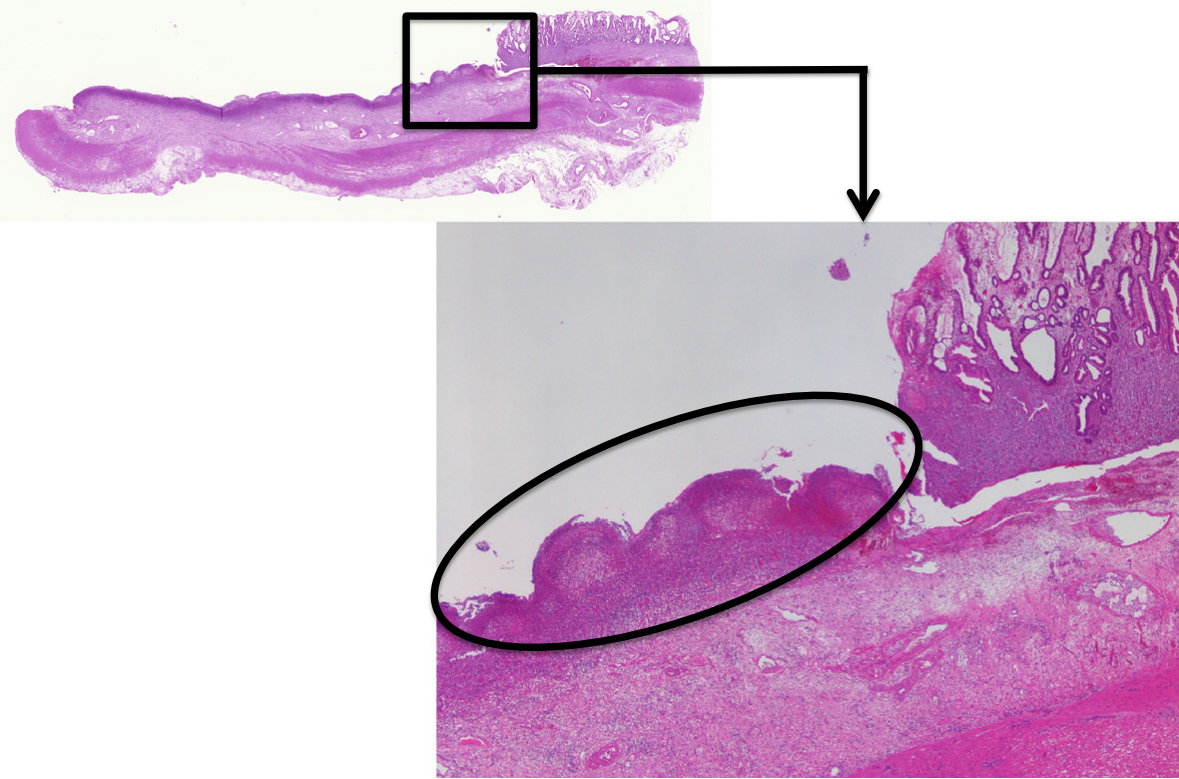
**Pathological findings within the small-bowel specimen.** Wide-ranging membrane exclusion and ulcer formation (black circle), and inflammation and fibrosis that extended from the sub-mucosal to the sub-serosal layers were observed. These pathological changes were associated with significant infiltration by a variety of inflammatory cells.

After undergoing the re-operation, the patient’s course was uneventful. However, 57 days after the initial operation, the patient experienced symptoms associated with a bowel obstruction. An upper gastrointestinal series revealed a small intestinal stenosis within the proximal jejunum. Fifty-nine days after the initial operation, a partial resection of the jejunum was performed. A severe luminal stricture of the jejunum was evident that was accompanied by marked structural changes, which were not apparent during the second operation (Figure 3). The pathological findings revealed an ischemic inflammation accompanied by an ulcer and necrosis. A wide-ranging exclusion of the membranes had occurred, with ulcer formation, and the inflammation and fibrosis reached the sub-mucosal and sub-serosal layers were observed (Figure 4). After the third operation, the patient’s course was uneventful, and he was discharged on an oral diet.

## Discussion

In a recent survey of HPVG, Kinoshita et al. reported a mortality rate of 39% among all 182 cases reported by 2001. HPVG occurs in different clinical scenarios, including bowel necrosis, digestive tract dilation, intraperitoneal abscesses, ulcerative colitis, Crohn’s disease, gastric ulcers, complications associated with endoscopic procedures, and intraperitoneal tumors. Most frequent cause of HPVG is mesenteric vascular occlusion and subsequent bowel necrosis. The mortality rate is high among cases of HPVG that is associated with bowel necrosis (75%), and emergency surgery is recommended for these potentially lethal cases [4]. Given the high mortality rate of HPVG associated with bowel necrosis, the underlying disease might be an important factor that contributes to patient survival. The degree of bowel necrosis also seems to be associated with the mortality rate. Although NOMI is an important pathology associated with HPVG and it has the highest mortality rates when it is associated with different critical conditions, an increasing number of patients managed using conservative treatment have been reported [11]. In 1958, Ende described the first cases of NOMI in 3 patients with heart-failure-associated low outputs [21]. Recently, a variety of predisposing factors for NOMI have been reported, including heart failure/cardiogenic shock, aortic insufficiency, the administration of vasoconstrictive medications, and dialysis [11,13,15]. Although a relatively low level of mortality has been demonstrated among dialysis patients without bowel necrosis, NOMI has the poorest survival rate among the different mesenteric ischemia etiologies, primarily because of the severity of the comorbid conditions that precipitate reductions in mesenteric perfusion and diagnostic delays. NOMI is associated with 20–30% of all cases of acute mesenteric ischemia and has a mortality rate of 70–90%, which has changed little over time, it is associated with 20–30% of all cases of acute mesenteric ischemia. It leads to extensive irreversible intestinal necrosis for which the prognosis is very poor, despite the absence of organic obstructions in the principal arteries. Contrast-enhanced CT scanning reveals patent mesenteric vessels, but the intestinal wall shows a reduced level of enhancement, both of which are indicative of a diagnosis of NOMI, and surgical intervention might be required if the ischemic damage progresses. In this case, we were unable to perform contrast-enhanced CT because the patient had acute kidney injury due to septic shock. However, portal venous gas secondary to NOMI was strongly suspected, because the patient had septic shock with diffuse peritonitis, extended pneumatosis intestinalis on CT, and no evidence of vascular occlusion with segmental circulatory insufficiency during surgical exploration. Pathological investigations of delayed strictures of the small bowel caused by ischemia have reported that the membranous exclusion and ulcer formation are caused by bowel perfusion disorders. Inflammation and fibrosis then reach the sub-mucosal and sub-serosal layers and extend to incorporate the entire circumference of the bowel lumen [22,23]. In the current case, the same pathological findings were observed, and these formed part of the rationale for our diagnosis of a delayed intestinal stricture caused by NOMI.

NOMI can be treated surgically or non-surgically. Surgical treatment should be considered for patients with suspected bowel necrosis or peritonitis, and for those whose laboratory investigations reveal leukocytosis, elevated levels of liver enzymes, creatinine phosphokinase, serum urea, creatinine, and amylase, and acidosis [10]. Presently, early angiography is performed on patients in whom mesenteric ischemia is suspected and there are no signs of sepsis or peritonitis. If NOMI without the evidence of bowel necrosis or peritonitis is diagnosed, vasodilators will be initiated, for example, prostaglandin E1 and nitroglycerin [11,13]. If gangrenous or transmural ischemic changes in the bowel are found during surgical exploration, resectional treatment is recommended. However, a bowel that is not infarcted but is of questionable viability should not be resected, and a second-look laparotomy is indicated in these cases [14]. The risks and benefits associated with resectional and non-resectional treatments are yet to be determined. While non-resectional treatment could avoid the complications associated with laparotomy and intestinal resection, a delayed intestinal stricture as occurred in this case, might be a risk associated with non-resectional treatment. Bowel stenoses that present after non-surgical treatment have been reported in patients with blunt abdominal trauma [16,17], inflammatory bowel disease, tuberculosis [18], ischemic colitis [19,20], and in non-steroidal anti-inflammatory drug-related enteropathy [24]. Pneumatosis that is accompanied by neonatal necrotizing enterocolitis has also been reported in 10–20% of the cases who present with delayed strictures [25-27]. A literature search performed on Medline (Ovid) and PubMed using the key words “non-occlusive mesenteric ischemia” and “stricture” or “stenosis”, from 1965 to 2013, uncovered only 2 cases [28], and no cases have been reported since the use of CT technology became routine.

Previously, NOMI was recognized as an acute phase critical condition that required early identification and appropriate surgical intervention. However, the possibility of delayed structural changes in the ischemic bowel of NOMI patients who are managed without resection of the involved bowel must be acknowledged, as has been demonstrated in the present case report.

## Conclusion

While non-surgical management can be considered for selected patients with NOMI, continuous observation is required to evaluate the development of delayed strictures that will lead to bowel obstructions in patients who undergo non-resectional treatment.

## Consent

Written informed consent was obtained from the patient for publication of this Case report and any accompanying images. A copy of the written consent is available for review by the Editor of this journal.

## Abbreviations

HPVG: Hepatic portal venous gas
CT: Computed tomography
NOMI: Non-occlusive mesenteric ischemia
ED: Emergency department
